# Supplementary material for: Alcohol Metabolism Enriches Squamous Cell Carcinoma Cancer Stem Cells That Survive Oxidative Stress via Autophagy
Source: Biomolecules. 2021 Oct 7;11(10):1479. doi: 10.3390/biom11101479 (PMC8533166; doi:10.3390/biom11101479)
Supplement: Supplementary file 1 [file biomolecules-11-01479-s001.zip › biomolecules-1280071-supplementary.pdf]

Supplementary Information

Alcohol metabolism enriches squamous cell carcinoma cancer stem cells that survive oxidative stress via autophagy

Masataka Shimonosono et al.

Supplementary Methods

Genotyping for ALDH2 single nucleotide polymorphism

G Genomic DNA was isolated from TE11, TE14, ESC2-3, and HSC1-3 lines using the DNeasy Blood and Tissue Kit (Qiagen, #69504). Genotyping for the ALDH2*2 single nucleotide polymorphism (rs671) was performed with the TaqMan drug metabolism genotyping assay (Thermo Fisher Scientific, #4351379) and TaqMan Genotyping Master Mix (Thermo Fisher Scientific, 4371353) using 10 ng of isolated genomic DNA per well. All reactions were carried out using the StepOnePlus Real-Time PCR System (Applied Biosystems). Analysis was performed with the Applied Biosystems Genotyping Analysis Module using default settings on Thermo Fisher Cloud according to manufacturer's instructions.

Immunoblot analysis

Immunoblot analysis of EtOH-exposed cells was performed as described previously (see reference [10] in the main text) utilizing anti-Phospho-p70 S6 Kinase (Thr389) (1:1000, #9205), anti-p70 S6 Kinase (1:1000, #9202), anti-Phospho-4E-BP1 (Ser65) (1:1000, #9451), anti-4E-BP1 (1:1000, #9452), and anti-β-Actin (1:1000, #4967) as primary antibodies. Densitometry of resulting signals was performed with Image Lab software (Bio-Rad). The signal intensity for molecule of interest was calibrated by that of β-actin.

Supplementary Table S1 ALDH2 SNP genotypes in ESCC cell lines and patient-derived organoid lines

Name	ALDH2 allele	Characterization
ESC2	ALDH2*1/ALDH2*1	Homozygous (wildtype)
ESC3	ALDH2*1/ALDH2*1	Homozygous (wildtype)
HSC1	ALDH2*1/ALDH2*1	Homozygous (wildtype)
HSC2	ALDH2*1/ALDH2*1	Homozygous (wildtype)
HSC3	ALDH2*1/ALDH2*1	Homozygous (wildtype)
TE11	ALDH2*1/ALDH2*2	Heterozygous (wildtype, SNP)
TE14	ALDH2*1/ALDH2*2	Heterozygous (wildtype, SNP)

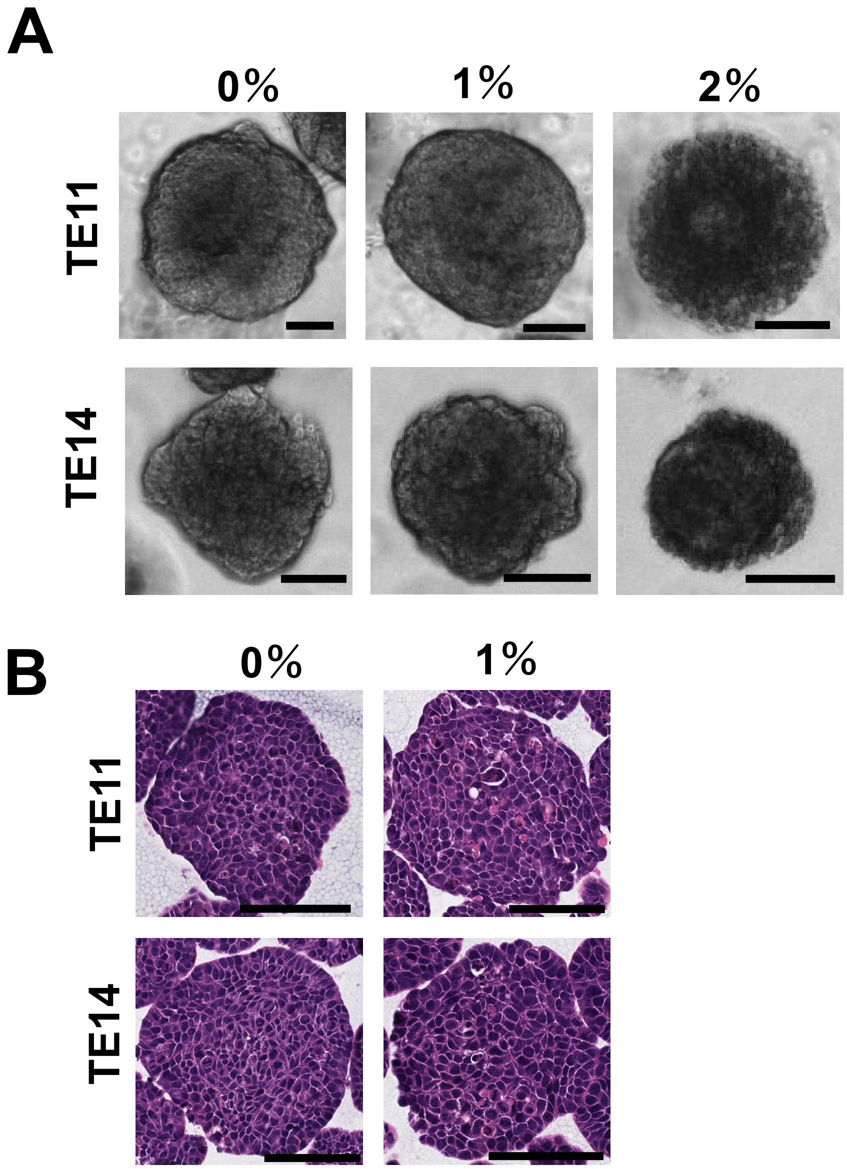

Supplementary Figure S1 1% EtOH maintains viable 1° organoid structure. 3D organoids generated with TE11 and TE14 cells were treated with or without EtOH at indicated concentrations for 4 days, starting from day 7. Phase-contrast images (A) and H&E staining images (B) of representative organoids are shown. Scale bar: 100 μm. Note that viable structures were recovered for H&E staining when organoids were treated with 2% EtOH in (B). There are many organoids with no or few apoptotic cells in no-treatment control while most organoids have many apoptotic cells after EtOH treatment. Note that phase-contrast images were more obscure in organoids treated with 2% EtOH in (A). Viable structures were not recovered when organoids were treated with 2% EtOH in (B), suggesting that 2% EtOH impaired cell viability more severely than 1% EtOH.

A

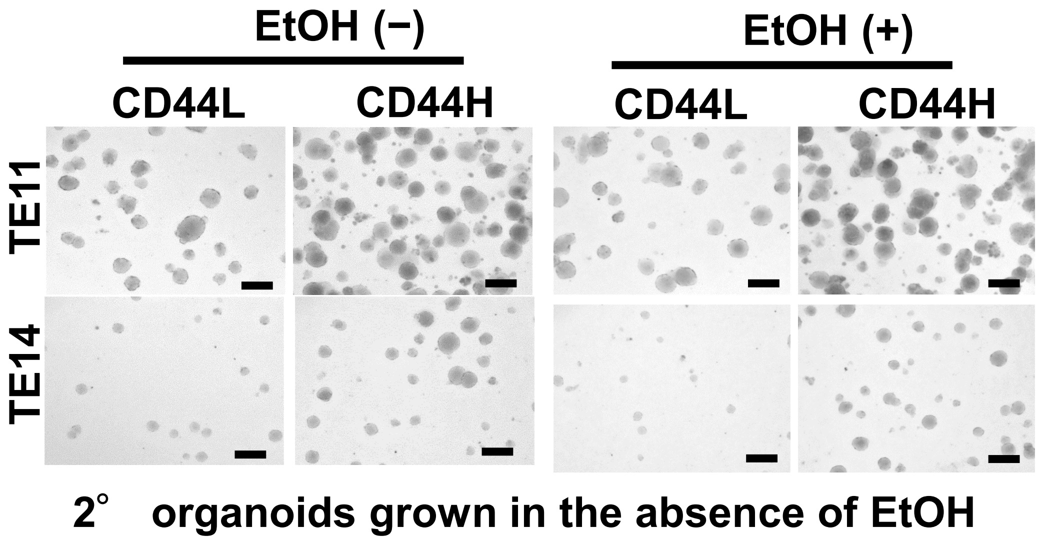

B

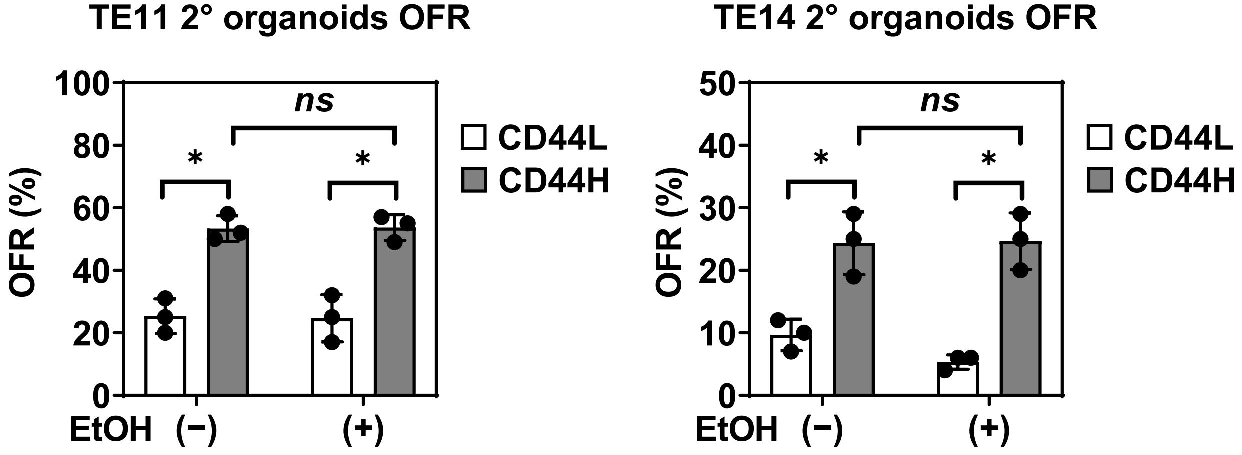

Supplementary Figure S2 Characterization of FACS-purified CD44L and CD44H cells isolated from primary organoids treated with or without EtOH (A, B) TE11 and TE14 organoids were treated with or without 1% EtOH for 4 days in 1° organoid. FACS-purified CD44L and CD44H cells of 1° organoids were passaged to grow 2° organoids in sub-culture (A). OFRs of 2° organoids were determined and plotted in bar graphs (B). Scale bar: 100 µm. *ns,* not significant vs. CD44H in EtOH (−); *, $p < 0.05$ vs. CD44L. n=3.

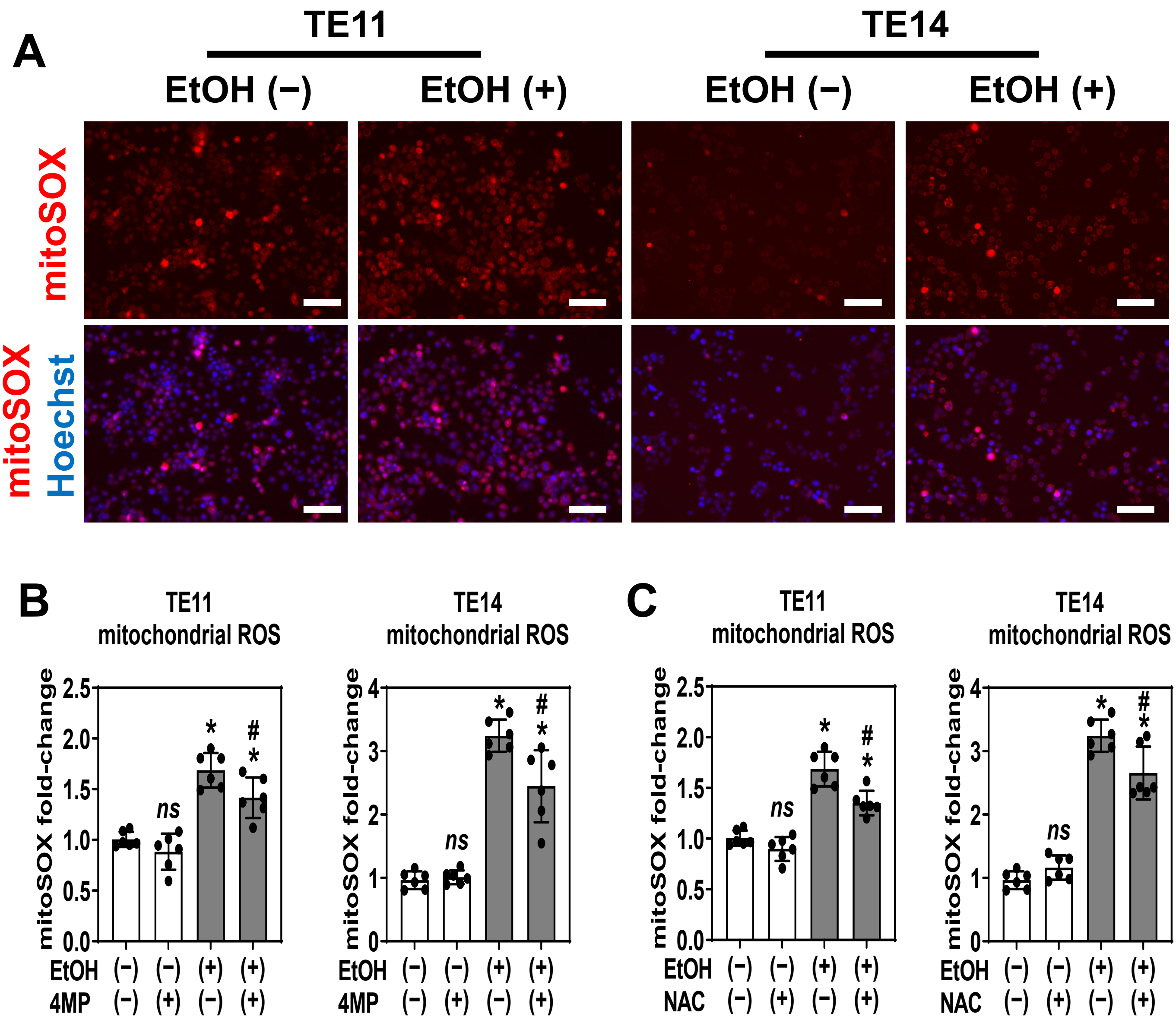

Supplementary Figure S3 ADH-mediated EtOH oxidation and oxidative stress are associated with increased mitochondrial superoxide production in EtOH-exposed SCC cells. TE11 and TE14 cells were cultured in monolayer culture for 24 hours and treated with 1% EtOH for 6 hours with or without 2 mM of 4MP treatment (B) or 10 mM of NAC pretreatment (C). Relative mitochondrial ROS levels were measured by Keyence microscope. (A) Scale bar: 100 µm. (B) *ns,* not significant vs. EtOH (−) and 4MP (−); *, $p < 0.05$ vs. EtOH (−) and 4MP (−); #, $p < 0.05$ vs. EtOH (+) and 4MP (−). n=6. (C) *ns*: not significant vs. EtOH (−) and NAC (−); *, $p < 0.05$ vs. EtOH (−) and NAC (−); #, $p < 0.05$ vs. EtOH (+) and NAC (−). n=6.

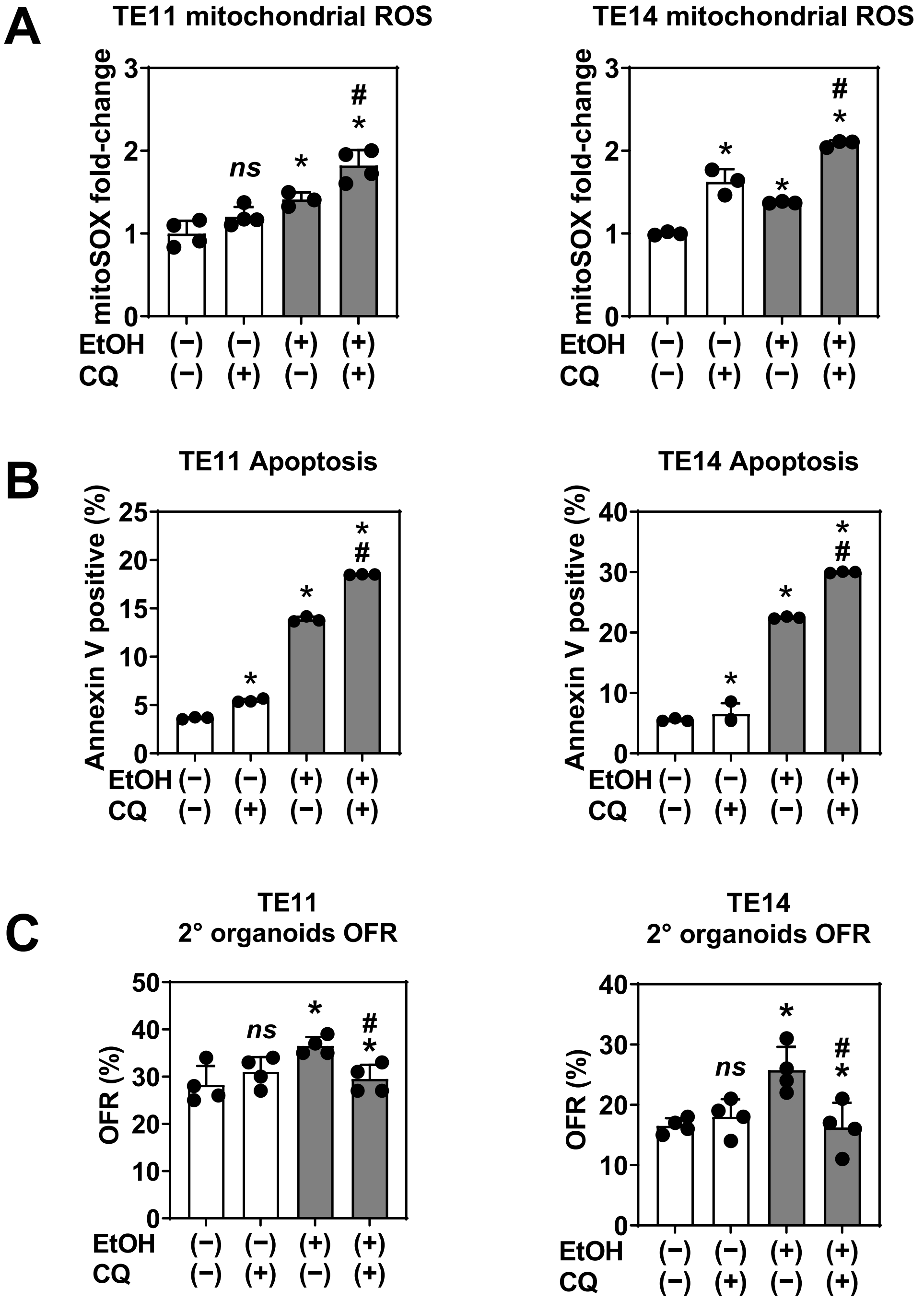

Supplementary Figure S4 Autophagy limits EtOH-induced mitochondrial superoxide production and apoptosis to mediate CD44H cell enrichment. (A) TE11 and TE14 cells were cultured in monolayer for 24 hours and treated with 1% EtOH for 6 h along with or without 2 μM CQ. Relative mitochondrial ROS levels were evaluated by Keyence microscope. (B, C) TE11 and TE14 organoids were treated with 1% EtOH for 4 days along with or without 2 μM CQ. Dissociated organoids were analyzed by Flow cytometry for Annexin V positive cells to determine apoptosis as shown in (B). 2° organoids were grown in the absence of EtOH and OFR was determined as plotted in graphs (C). *ns*, not significant vs. EtOH (−) and CQ (−); *, $p < 0.05$ vs. EtOH (−) and CQ (−); #, $p < 0.05$ vs. EtOH (+) and CQ (−). n=4 in (A) TE11 and (C), n=3 in (A) TE14 and (B).

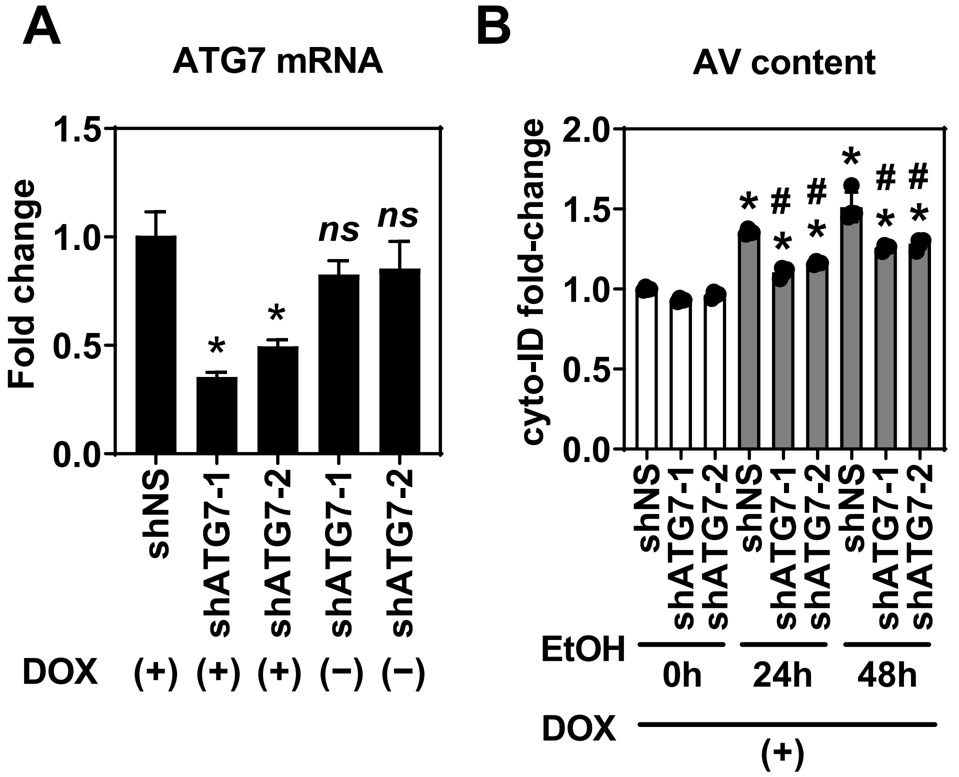

Supplementary Figure S5 ***ATG7* knockdown by shRNA decreases EtOH-induced autophagy in SCC cells.** (A, B) TE11 cells with indicated genotypes were treated with or without DOX for 48 h to induce shRNA in monolayer culture. Without DOX treatment, shRNA did not affect *ATG7* mRNA expression levels or ATG7 protein expression levels. (A*)* *ATG7* mRNA expression levels were confirmed by qPCR. *, $p < 0.05$ vs. shNS. n=3. (B) TE11 cells with indicated genotypes were treated with 1% EtOH along with DOX in monolayer culture. Harvested cells were analyzed by flow cytometry to determine the AV content. *, $p < 0.05$ vs. shNS in 0 h EtOH; #, $p < 0.05$ vs. shNS in 24 h or 48 h EtOH treatment. n=3.

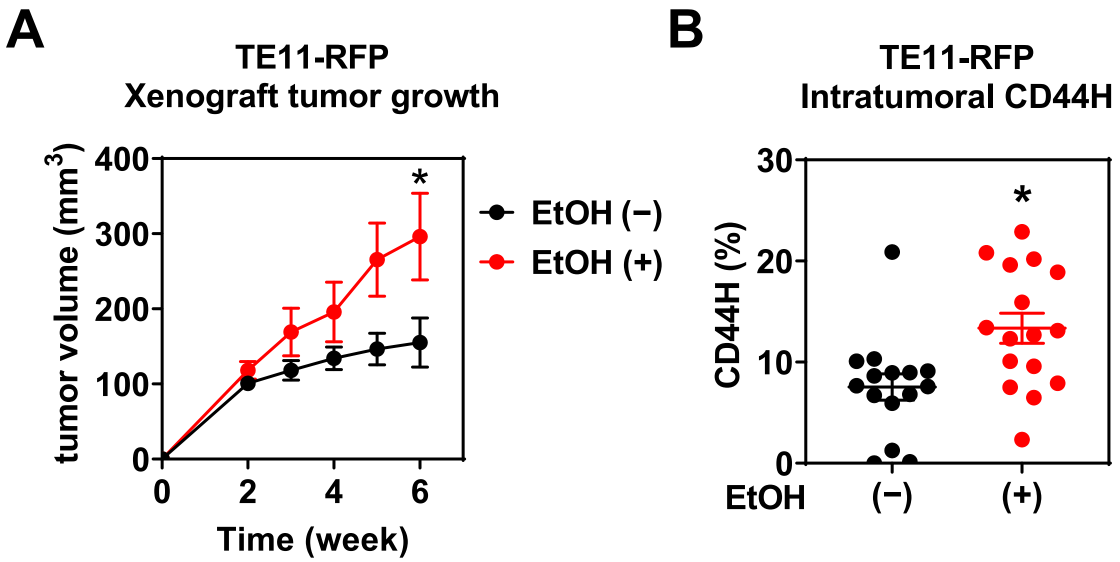

Supplementary Figure S6 EtOH promotes xenograft tumor growth in alcohol-fed immunodeficient mice. TE11-RFP cells were subcutaneously injected to the lower back of immunodeficient mice. Mice were given 10% EtOH in drinking water for 4 weeks, starting 2 weeks after tumor cell implantation. Tumor volume was measured once a week and plotted in graphs. (A) *, $p < 0.05$ vs. EtOH (−), n=16/group. (B) Harvested tumors were dissociated and analyzed by flow cytometry to determine intratumoral CD44H cells. *, $p < 0.05$ vs. EtOH (−). n=15 for EtOH (−). n=16 for EtOH (+).

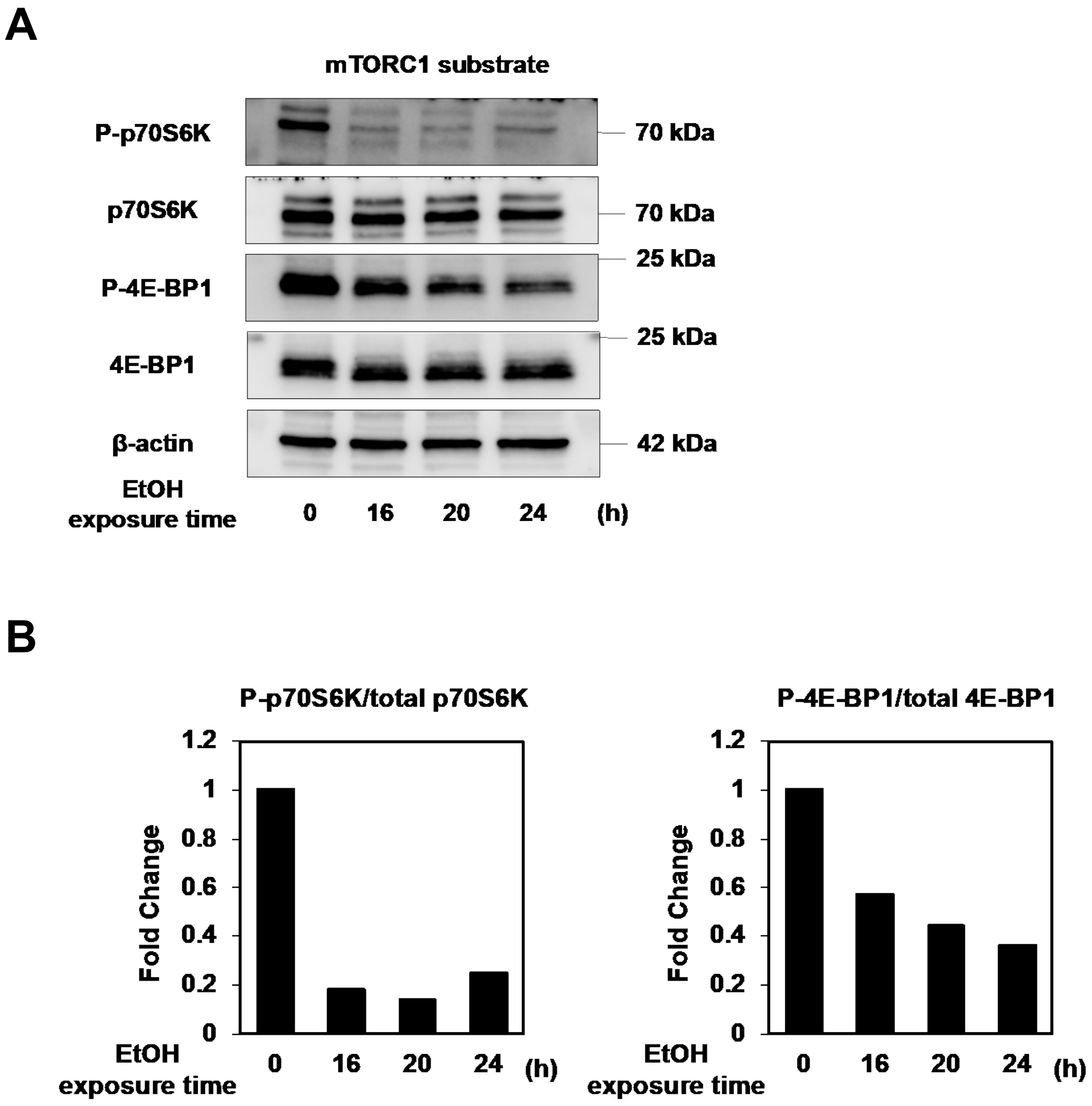

Supplementary Figure S7 EtOH may suppress mTORC1 signaling in TE14 cells. (**A and B**) TE14 cells were treated with 1% EtOH for indicated time periods and subjected to immunoblot analysis to determine phosphorylation of mTORC1 substrates p70 S6 kinase (p70 S6K) and 4E-BP1 proteins with β-actin serving as a loading control. Densitometry determined phosphorylation of p70 S6K (P-p70 S6K) and 4E-BP1 (P-4E-BP1) relative to total p70 S6K and 4E-BP1 proteins, respectively, in (**B**). Immunoblots represent one of two independent experiments with similar results. See **Supplementary Figure S8** for uncropped immunoblot images.

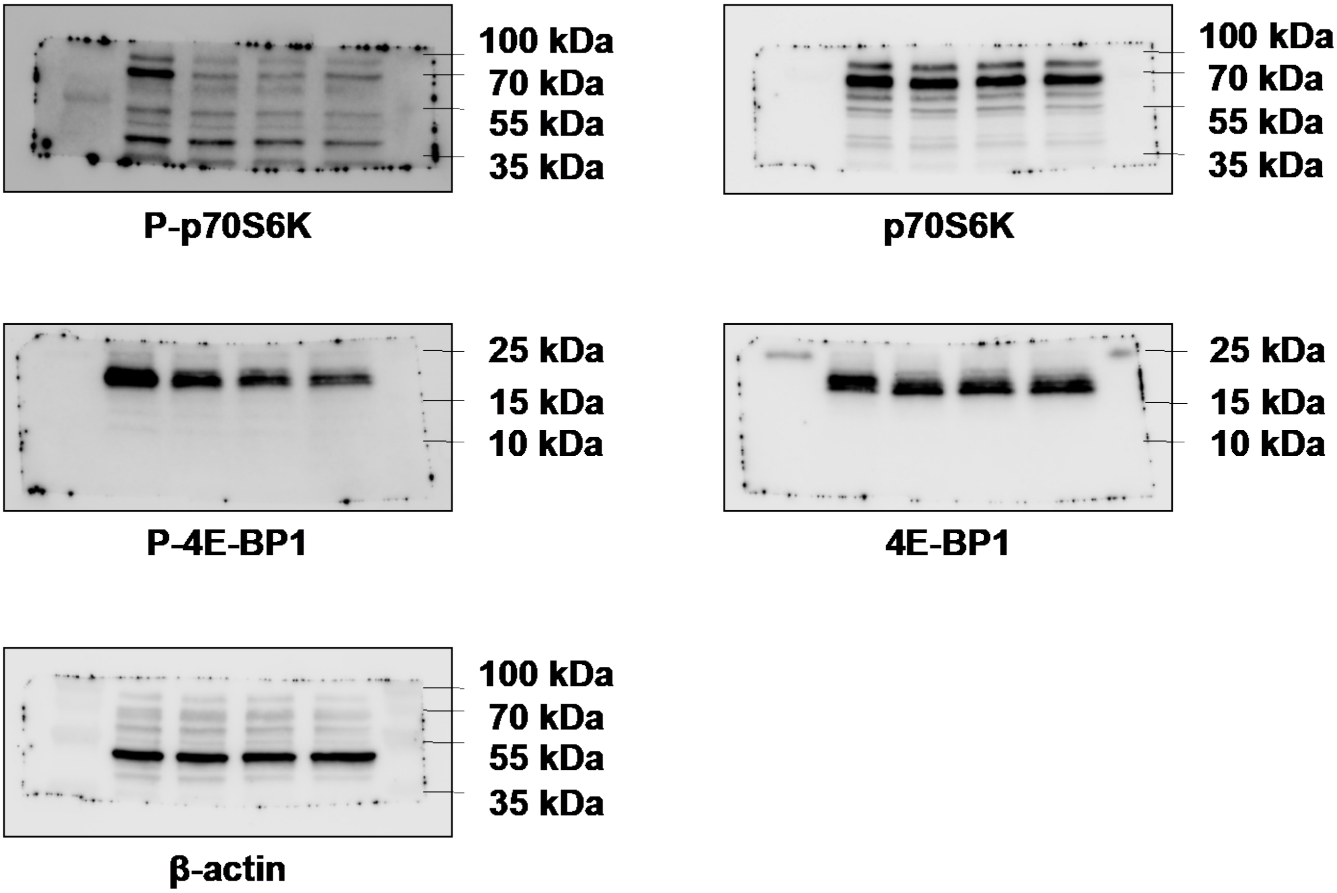

Supplementary Figure S8 Un cropped immune blot images corresponding to Supplementary Figure S7
